# Antibody-Radionuclide Conjugates for Solid Tumors: Multidimensional Strategies from Component Engineering to Synergistic Combination Therapy

**DOI:** 10.3390/ijms27188022

**Published:** 2026-09-09

**Authors:** Mohan Zhao, Xiaojun Zhao, Zixiang Gao, Rongguang Shao, Li Liu, Wuli Zhao

**Affiliations:** State Key Laboratory of Respiratory Health and Multimorbidity, Key Laboratory of Antibiotic Bioengineering, Ministry of Health, Bejing Key Laboratory of Long-Acting Targeted Delivery Technology and Applications for Peptide and Nucleic Acid Drugs, Laboratory of Oncology, Institute of Medicinal Biotechnology, Chinese Academy of Medical Sciences & Peking Union Medical College, Beijing 100050, China; s2025010028@student.pumc.edu.cn (M.Z.); zhaoxjun00@163.com (X.Z.); gzx120539232@163.com (Z.G.); rongguangshao@163.com (R.S.)

**Keywords:** antibody-radionuclide conjugates, solid tumor, combination therapy, pretargeting, translational barriers

## Abstract

Antibody-radionuclide conjugates (ARCs), defined as systems in which antibodies or their derivatives are linked to therapeutic or diagnostic radionuclides via covalent or non-covalent strategies, represent a promising platform for precision tumor theranostics. Their clinical application in solid tumors, however, remains constrained by poor tumor penetration, heterogeneous antigen expression, off-tumor toxicity, and an immunosuppressive tumor microenvironment. To systematically address these barriers, multidimensional strategies are being pursued. Structurally, innovations in radionuclide selection, antibody engineering, and chelator chemistry are enhancing targeting efficacy and in vivo stability. Strategically, pretargeting approaches decouple antibody localization from radionuclide delivery to minimize off-target exposure. Systematically, matched theranostic pairs enable image-guided patient stratification and personalized dosimetry. Biologically, combining ARCs with immune checkpoint inhibitors or DNA damage response inhibitors remodels the tumor microenvironment and converts localized radiation into systemic antitumor immunity. This review critically evaluates recent advances across these domains, from component optimization, pretargeting strategies, and theranostic integration, to combination regimens, and highlights the translational barriers that must be overcome to realize the full clinical potential of ARCs in solid tumors.

## 1. Introduction

Chemotherapy remains a cornerstone of cancer treatment, yet its effectiveness against solid tumors is frequently constrained by inherent tumor characteristics such as invasiveness and metastatic potential, in addition to the development of drug resistance [1,2]. Radiopharmaceutical therapy offers unique targeting capability and high diagnostic sensitivity, but conventional techniques frequently fail to accurately target micrometastases, leading to off-tumor toxicity that restricts therapeutic efficacy in solid tumors [3,4]. These limitations highlight the need for more precise and effective treatment modalities.

ARCs are theranostic agents that pair tumor-targeting antibodies or fragments with radionuclides. Unlike small-molecule radioligand therapies that target prostate-specific membrane antigen (PSMA), such as [^177^Lu]Lu-PSMA-617, ARCs leverage both directly conjugated and pretargeted linkage strategies to deliver radiation selectively to tumor cells, enabling diagnostic imaging for patient selection and subsequent therapeutic application.

It is instructive to position ARCs within the broader landscape of antibody-based therapeutics, particularly compared with antibody-drug conjugates (ADCs). Agents such as trastuzumab (Herceptin) have achieved notable clinical success in HER2-positive cancers, yet their efficacy is frequently constrained by antigen expression heterogeneity and acquired resistance [5]. ARCs address this limitation through a fundamentally different mechanism: rather than relying on internalization and intracellular payload release, they deliver ionizing radiation predominantly extracellularly, inducing DNA damage in both antigen-positive and neighboring antigen-negative cells via bystander and crossfire effects (particularly for β-emitting radionuclides), thereby overcoming the barrier of heterogeneous antigen expression. Moreover, the theranostic nature of ARCs—enabled by matched radionuclide pairs—offers a capability not available to ADCs [6,7].

Despite these mechanistic advantages, first-generation ARCs, initially developed for hematological malignancies, targeted a range of antigens—including CD20 (e.g., ^90^Y-ibritumomab tiuxetan), CD33, and CD45—and demonstrated the clinical feasibility of antibody-based radionuclide delivery [8,9,10]. However, their translation to solid tumors has been hampered by poor tissue penetration due to the large size of antibodies (~150 kDa), heterogeneous antigen expression, and the immunosuppressive tumor microenvironment (TME), often resulting in suboptimal efficacy and dose-limiting toxicities such as bone marrow suppression [11].

To overcome the barriers mentioned above and enhance the precision, efficacy, and safety of ARC therapy, multidimensional strategies are being actively pursued. Structurally, selecting α-emitters, miniaturized or bispecific antibodies (BsAbs), and advanced bifunctional chelators can improve targeting, pharmacokinetics, and stability. Strategically, pretargeting approaches decouple antibody targeting from radiation delivery to reduce normal tissue exposure and nonspecific damage. Systematically, employing matched theranostic pairs enables patient-specific dosimetry and treatment monitoring, improving therapeutic accuracy. Synergistically, combining ARCs with immunotherapies or DNA damage repair inhibitors (DDRis) addresses limitations imposed by tumor heterogeneity and the immunosuppressive microenvironment, enhancing precise tumor killing, remodeling the TME, and inducing sustained antitumor immunity.

Given the promising potential of ARCs in solid tumor therapy, this review systematically examines the evolution of ARCs from component optimization and delivery innovations to combination regimens. We critically evaluate how these advances are transforming ARC technology from a localized treatment tool into a multifunctional platform for systemic, precision oncology in solid tumors. By synthesizing recent preclinical and clinical progress, we highlight ARC strategies that hold promise for overcoming longstanding therapeutic bottlenecks and improving outcomes for patients with advanced solid malignancies.

## 2. Optimizing ARCs Architecture for Enhanced Targeting and Efficacy

**Figure 1:**
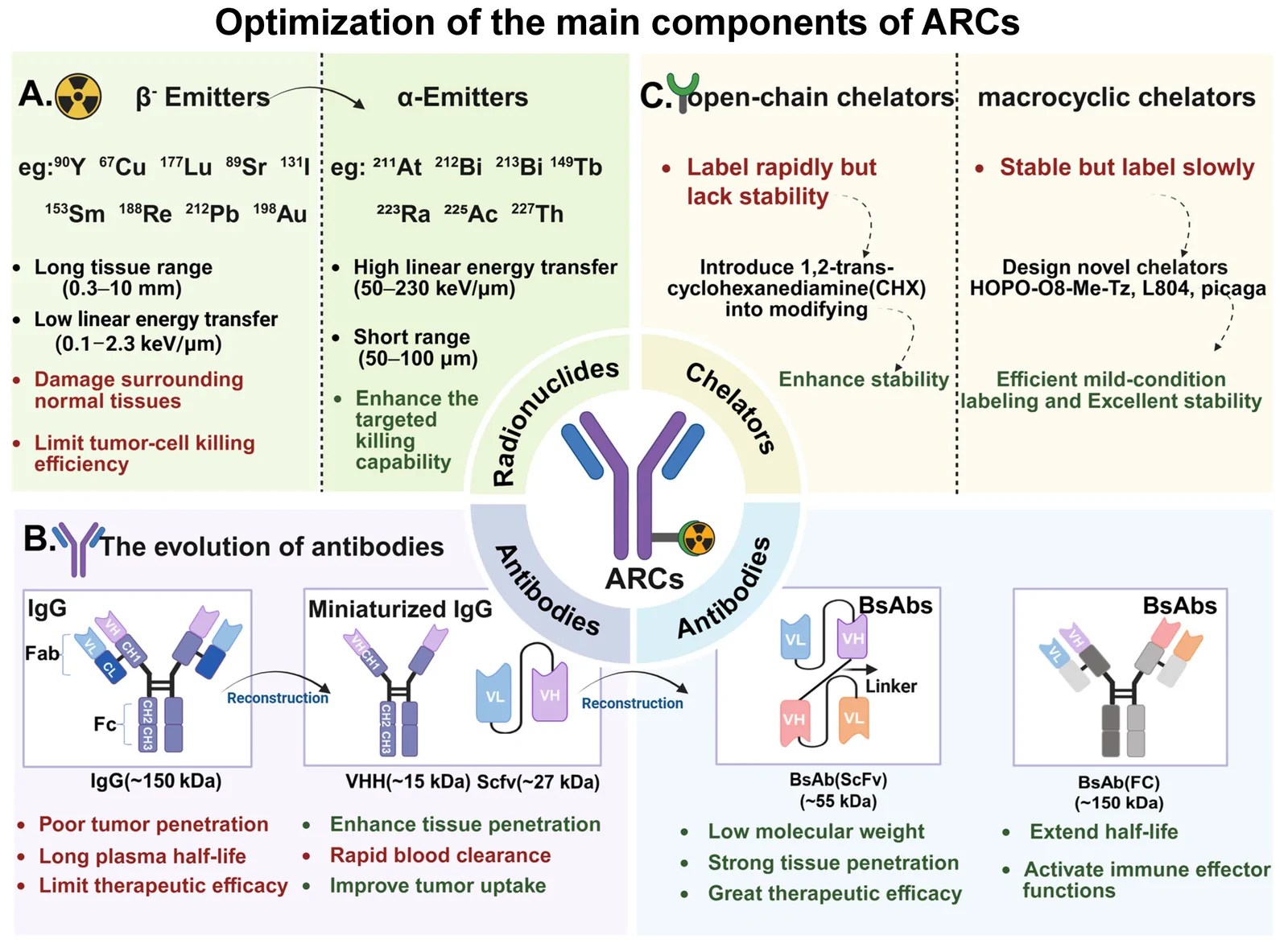
Optimization of the main components of ARCs. (**A**) Classification and characteristics of therapeutic radionuclides, illustrating the research hotspot shift from β-emitters to α-emitters, with key translational barriers noted (supply, dosimetry, daughter redistribution). (**B**) Structural modification of IgG into miniaturized antibodies and BsAbs to overcome size-related penetration obstacles, with trade-offs in half-life and retention highlighted. (**C**) Modification of open-chain and macrocyclic chelators to achieve rapid room-temperature labeling and high in vivo stability. Created in BioRender. mohan, Z. (2026) https://BioRender.com/ivb0aq4.

Through the synergistic action of their components, ARCs achieve targeted treatment by specifically binding to tumor-associated antigens, thereby concentrating the radionuclide at the tumor site. A targeting antibody delivers the complex to antigens on the tumor cell surface, a bifunctional chelator ensures radionuclide stability in systemic circulation, and the decaying radionuclide induces lethal DNA damage to kill tumor cells. Optimizing each component is crucial for improving tumor selectivity and reducing off-target effects (Figure 1).

### 2.1. Research Hotspot Shifting from β- to α-Emitters: The Move Toward High-LET Radiation

The radionuclide serves as the effector component of ARCs, emitting β-particles or potent α-particles to induce irreversible DNA damage in targeted tumor cells. β-emitters (e.g., ^177^Lu, ^131^I) exhibit relatively long tissue range (0.3–10 mm) and low linear energy transfer (LET, 0.1–2.3 keV/μm), which can damage surrounding normal tissues and limit tumor-cell killing efficiency [12]. Moreover, their cytotoxicity is influenced by factors within the TME, such as cell metabolism and oxygen levels, further restricting their application in solid tumors [13]. In recent years, growing research interest has been directed toward α-emitting radionuclides (e.g., ^223^Ra, ^225^Ac,^211^At) for targeted therapy, driven by their high linear energy transfer (LET) (50–230 keV/μm) and short tissue range (50–100 μm) [14,15]. Their cytotoxicity is independent of cell cycle phase and oxygen concentration, making them particularly suitable for hypoxic tumors [16]. Although free α-emitters can cause significant hematologic and organ toxicity, conjugation to targeting vehicles confines radiation to tumor sites, improving the therapeutic index [17,18]. Preclinical studies in prostate, pancreatic, and melanoma models have confirmed the potent antitumor activity of α-emitting ARCs, positioning them at the forefront of next-generation radiopharmaceutical development [19,20,21,22] (Figure 1A).

Notwithstanding these radiobiological advantages, the clinical translation of α-emitting ARCs is confronted by four interconnected hurdles that are often overshadowed by their therapeutic promise. First, α-recoil-driven redistribution of daughter nuclides causes off-target accumulation in kidneys, liver, and bone marrow, undermining therapeutic selectivity [23]. Second, global ^225^Ac production currently meets the needs of only a few thousand patients annually, while the 7.2 h half-life of ^211^At restricts its use to cyclotron-accessible regions [24]. Third, clinically validated microdosimetry frameworks for α-emitters remain lacking, hampering individualized dose optimization [25]. Fourth, long-term toxicity, particularly delayed bone marrow effects, remains poorly characterized [14]. Overcoming these obstacles requires coordinated advances in chelator chemistry, scalable isotope production, dosimetric standardization, and long-term follow-up from prospective clinical trials.

### 2.2. Antibodies: Balancing Specificity, Penetration, and Pharmacokinetics

Antibodies, with their high affinity and specificity for antigens, serve as ideal carriers for targeted radionuclide delivery. However, their clinical application faces two major challenges. First, the large molecular size of intact monoclonal antibodies (IgG, ~150 kDa) results in poor tumor penetration and a long plasma half-life, limiting therapeutic efficacy in solid tumors. Second, the targets recognized by antibodies often lack highly specific distribution on tumor cell surfaces, which can trigger off-target effects and cytotoxic adverse reactions. To address these limitations, strategies such as antibody miniaturization, development of BsAbs, and exploration of novel, highly specific tumor targets offer new therapeutic avenues (Figure 1B).

#### 2.2.1. Miniaturized Antibodies

To improve solid tumor penetration, researchers have developed various smaller antibody fragments that retain antigen-binding specificity while offering enhanced tissue distribution. These include Fab (~50 kDa) and F(ab′)_2_ (~110 kDa), which are proteolytically derived fragments of full-length IgG; single-chain variable fragments (scFv) (~28 kDa), composed of linked VH and VL domains; diabodies (~55 kDa) and triabodies (~80 kDa), which are multimeric scFv derivatives with enhanced avidity; and nanobodies (also known as VHH, ~15 kDa), which are single-domain antigen-binding fragments derived from camelid heavy-chain antibodies. Among these, scFv and VHH have been the most extensively explored for ARC applications. These miniaturized formats retain the targeting specificity of full-length IgG while offering superior pharmacokinetics, enhanced tissue penetration, and rapid blood clearance that improve tumor uptake and enable new diagnostic and therapeutic applications [26,27,28]. Currently, miniaturized antibodies enable highly sensitive PET and SPECT imaging in gastric, breast, and other solid tumor models, providing accurate lesion localization and target expression assessment that address efficacy heterogeneity [29,30,31]. When conjugated to radionuclides, these fragments combine excellent tumor penetration with potent cytotoxicity, enabling precise radiation targeting [32,33,34]. Thus, from diagnostic identification to targeted therapy, miniaturized antibodies offer vital support for theranostics, helping to tackle the challenges posed by solid tumor heterogeneity.

#### 2.2.2. BsAbs

BsAbs represent an advanced approach in precision tumor therapy by enabling dual antigen targeting with a single molecule, thereby enhancing specificity and reducing off-target effects. Based on molecular architecture, BsAbs are broadly categorized into two classes [35]. One type consists of two or more antibody fragments (e.g., scFv), characterized by low molecular weight and strong tissue penetration, which is advantageous for solid tumors. The other retains the Fc region, which, while extending half-life, can activate immune effector functions such as antibody-dependent cellular cytotoxicity, offering dual advantages in pharmacokinetics and immune regulation, although the pharmacological relevance of this effect at ARC-relevant doses remains to be established. This versatility has spurred significant clinical development, with over 200 BsAbs in trials, approximately 75% for solid tumors [36,37]. Furthermore, the development of bispecific antibody-drug conjugates (ADCs) expands the therapeutic arsenal and provides a rationale for exploring BsAbs conjugated with radionuclides for solid tumor therapy [36].

Nevertheless, each of these antibody formats carries inherent limitations that temper their translational potential. Miniaturized formats improve penetration but suffer from reduced domain stability and manufacturing constraints linked to camelid origins [38]. More fundamentally, enhanced tissue penetration comes at the cost of shortened tumor retention. Lower molecular weight facilitates extravasation but leads to blood clearance within hours, potentially limiting the total radiation dose delivered to the tumor [39]. Bispecific antibodies face a different set of complexities: proper folding and assembly of two distinct binding domains, precise affinity tuning to balance efficacy against off-target toxicity, structure-dependent pharmacokinetic optimization, and manufacturing complexity coupled with immunogenicity risks from non-native architectures [37,40]. Current research addresses these issues through three complementary directions: smart linker engineering, optimal format selection, and exploitation of differential antigen expression.

#### 2.2.3. Novel Target Antibodies

Leveraging the exclusive expression of genuine tumor-specific antigens on malignant cells, antibodies targeting these molecules offer a promising strategy to improve the selectivity of ARCs for solid tumors. These targets fall into two mechanistic classes. The first class comprises proteins that become selectively accessible in tumors due to disrupted tissue architecture. Claudin 18.2 (CLDN18.2) is a paradigmatic example: normally sequestered within gastric tight junctions, it becomes exposed on tumor cell surfaces, enabling specific antibody recognition with minimal off-target toxicity [41]. In the Phase I KYM901 trial of the CLDN18.2-targeting ADC CMG901, the confirmed objective response rate (cORR) in the total patient population (n = 89) was 33% (95% CI, 23.0–43.3%), and the confirmed disease control rate (DCR) was 70% (95% CI, 59.0–79.0%); in a subset of eight patients with high CLDN18.2 expression, the ORR reached 75% and DCR 100% [42].

The second category comprises structurally distinct antigen variants generated exclusively by tumors, including EGFRvIII, cleaved Trop-2, and glycosylated PD-L1. Their absence from normal tissues makes them ideal for achieving absolute tumor selectivity. Antibodies targeting these variants have shown promising preclinical efficacy, with EGFRvIII-targeting agents extending progression-free survival to 6.1 months in patients and glycosylated PD-L1-targeting antibodies inducing complete remission in 70% of triple-negative breast cancer mouse models [43,44,45].

It should be noted that the agents discussed above, including CMG901, anti-EGFRvIII, and anti-glycosylated PD-L1 antibodies, are antibody-drug conjugates or naked antibodies rather than ARCs. Nonetheless, their clinical and preclinical data validate the biological relevance of these highly tumor-selective targets. This validation supports repurposing these antigens for future ARC designs, where their selectivity advantages could improve therapeutic indices.

These results underscore the potent and specific antitumor activity of such antibodies, providing a strategic foundation for developing next-generation ARCs. These novel constructs could mitigate dose-limiting toxicities associated with broadly expressed targets, opening a more precise therapeutic avenue for solid tumors.

### 2.3. Bifunctional Chelators

Bifunctional chelators are critical linkers that connect targeting antibodies and radionuclides. Their performance dictates the safety and efficacy of ARCs. Conventional chelators face a fundamental trade-off: open-chain chelators (e.g., DTPA, DFO) label rapidly but lack stability, while macrocyclic ones (e.g., DOTA, NOTA) are stable but label slowly [46]. Both struggle to simultaneously achieve rapid room-temperature labeling and high in vivo stability, limiting the construction of stable ARCs and ultimately their therapeutic efficacy and safety [46]. Therefore, developing novel high-performance chelators is key to advancing next-generation ARCs (Figure 1C).

To overcome these limitations, modifications and innovations have been pursued in two structural categories. Introducing rigid modifications, such as 1,2-trans-cyclohexanediamine, into open-chain chelators can enhance stability without sacrificing kinetics. For instance, the H2CHXhox derivative of H2hox enables efficient labeling within 1 min at 25 °C and maintains strong stability even under strong acid conditions (pH = 1) [47]. CHX-A-DTPA exhibits a 150-fold improvement in stability over DTPA, ensuring radionuclide stability during in vivo circulation [48]. Furthermore, next-generation open-chain chelators like H4octapa can achieve 98% radiolabeling yield for nuclides (e.g., ^111^In, ^177^Lu) within 10 min, with in vivo stability comparable to DOTA [49].

Novel macrocyclic chelators (e.g., HOPO-O8-Me-Tz, L804, picaga) are designed for efficient, mild-condition labeling with excellent stability and tumor targeting/uptake performance, making them ideal for constructing next-generation ARCs. Their superiority is demonstrated in related studies: L804 achieved >99% radiochemical yield versus DOTA [32]; HOPO-O8 complexes maintained >95% integrity over 7 days in vitro with tumor-to-bone ratios >11 in vivo [50]; and 44Sc-picaga-DUPA showed an 83% yield and 13.8% ID/g tumor uptake in mice [51]. Collectively, these advances in labeling kinetics, thermodynamic stability, and in vivo targeting performance address key bottlenecks in ARC development.

Beyond these conventional scaffold modifications, the chelator field has expanded into platforms tailored for theranostic applications. HOPO-type ligands (e.g., THP, HOPO-O8-Me-Tz) exhibit picomolar to nanomolar affinity for tri- and tetravalent metals (e.g., Zr^4+^, Tb^3+^, Th^4+^), enabling rapid, quantitative radiolabeling at room temperature with exceptional in vitro and in vivo stability [46,52,53,54,55]. Their broad specificity for radionuclides such as ^68^Ga, ^89^Zr (diagnostic) and ^177^Lu, ^225^Ac, ^212^Pb (therapeutic) makes them versatile platforms for constructing matched pairs. Dedicated dual-function chelators further expand this toolkit. The D2 chelator contains distinct metal-binding domains—DFOB for ^89^Zr and DOTA for ^177^Lu—allowing the same antibody conjugate to be radiolabeled with either ^89^Zr for PET imaging or ^177^Lu for therapy, ensuring matched pharmacokinetics for both applications [56]. Similarly, derivatives of the natural siderophore desferrioxamine B have been engineered to efficiently complex both ^68^Ga and therapeutic isotopes like ^177^Lu and ^225^Ac [57].

### 2.4. Specific Activity: A Critical Determinant of ARC Performance and Strategies for Improvement

The effective specific activity of an ARC, defined as the radioactivity per unit mass of the conjugate, affects tumor targeting, biodistribution, and therapeutic index [14,58]. For intact IgG-based constructs, achieving high specific activity is not trivial. Attaching multiple chelators can compromise antigen binding, increase heterogeneity, or reduce stability [59,60]. A separate issue is the presence of nonradioactive antibody in the final preparation, which competes with the radiolabeled species for antigen binding and lowers the actual radioactivity delivered per gram of tumor [14,58]. This problem is most pronounced when antigen density is low or heterogeneous, or when tumor perfusion is poor.

Several strategies have been explored to increase payload while preserving targeting function. Poly-L-lysine and other polycationic carriers provide additional chelator attachment sites, and dendritic or nanostructured systems (liposomes, polymeric nanoparticles, gold nanoparticles) have also been examined as platforms for delivering multiple payloads per targeting unit [61,62,63]. These modifications, however, introduce trade-offs. Increased hydrodynamic size, altered surface charge, enhanced liver/spleen uptake, and reduced tumor penetration have all been observed. For nanoparticles, rapid sequestration by the mononuclear phagocyte system and heterogeneous intratumoral distribution further limit the payload that reaches tumor cells. What matters clinically is the activity delivered and retained per unit of administered mass, not the nominal payload per carrier [62].

A high-payload construct with poor penetration may deliver less activity than a lower-payload construct with better biodistribution. Similarly, high specific activity is of little use if much of the conjugate is inactive or if the radionuclide half-life does not match antibody kinetics. Thus, specific-activity optimization should be part of a system-level design strategy. Reporting practices also remain inconsistent across studies, particularly for parameters such as activity per mass of antibody, chelator-to-antibody ratio, immunoreactive fraction, and labeled versus unlabeled fraction. More standardized reporting would help determine whether higher loading actually improves tumor dose delivery.

## 3. Pretargeted Radioimmunotherapy: Minimizing Nonspecific Exposure

The therapeutic efficacy of conventional ARCs in solid tumors is constrained by suboptimal tumor-to-background ratios, which result in inadequate tumor irradiation and dose-limiting normal-tissue toxicities. Pretargeted radioimmunotherapy (PRIT) has been proposed as an attractive strategy to address this limitation by temporally decoupling antibody localization from radionuclide delivery [64]. However, this reduction in normal-tissue exposure comes at the cost of lower absolute tumor uptake compared with directly labeled ARCs. The multi-step nature of PRIT, which requires optimization of multiple components and, in some cases, additional clearing steps, adds considerable complexity to clinical translation. In this multi-step approach, an unlabeled targeting agent (e.g., an antibody or scaffold protein) is administered first and allowed to accumulate at the tumor. After an optimal interval for blood clearance, a small-molecule radionuclide carrier designed for rapid clearance and specific binding to the pre-localized agent is injected, achieving high tumor radiation doses while minimizing systemic exposure [65].

**Figure 2:**
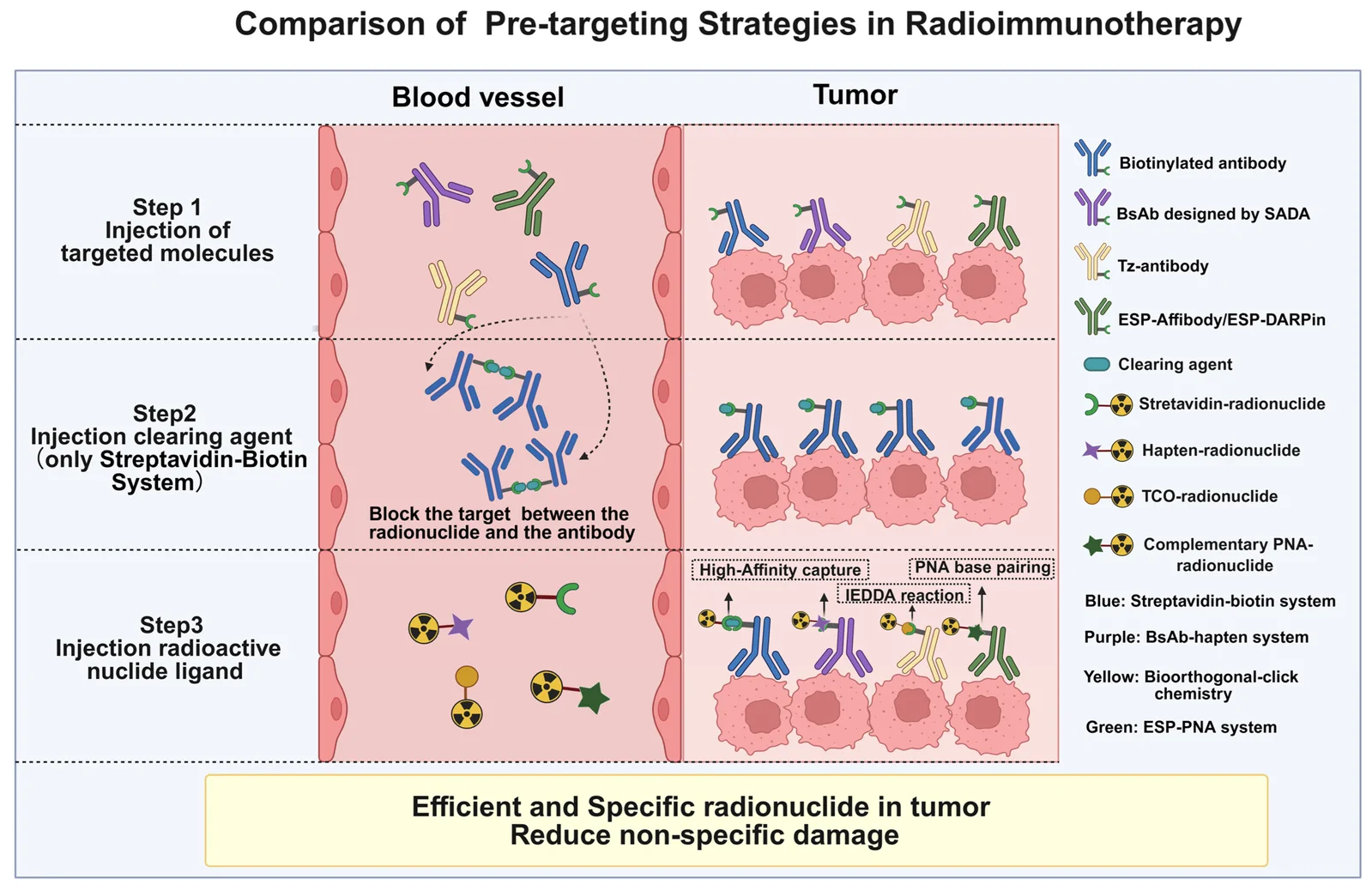
Mechanism diagrams of four pretargeting radioimmunotherapy strategies. The streptavidin-biotin system requires a clearing agent step, while the other three (BsAb-hapten/SADA, IEDDA click chemistry, ESP-PNA) do not. Created in BioRender. mohan, Z. (2026) https://BioRender.com/6wzh5if.

The pioneering streptavidin–biotin system, leveraging the ultra-high affinity (Kd~10^−15^ M) between streptavidin and biotin, first validated PRIT efficacy in clinical imaging and therapy [66]. However, its clinical utility in solid tumors has been constrained by several limitations: irreversible binding necessitating complex clearing protocols, nonspecific organ retention, and immunogenicity [67,68]. Consequently, recent efforts have focused on more sophisticated platforms, including bispecific antibody–hapten systems, bioorthogonal click chemistry, and engineered scaffold protein–peptide nucleic acid (ESP-PNA) hybrids, each offering distinct advantages (Figure 2).

### 3.1. BsAb-Hapten System

The BsAb-hapten system represents a major advancement in PRIT. In this strategy, a BsAb with one arm targeting a tumor antigen and the other a small-molecule hapten is administered first. After tumor localization and blood clearance, a radiolabeled hapten is injected, which is rapidly captured at the tumor site. This decouples targeting from delivery, achieving high tumor-specific radiation doses with minimal systemic exposure, thereby addressing key limitations of conventional ARCs such as poor tumor penetration and on-target, off-tumor toxicity.

Early BsAbs developed using the Dock-and-Lock platform enabled high-sensitivity PET imaging in breast cancer, preliminarily validating this strategy for solid tumor theranostics [69]. However, monovalent hapten binding and the need for complex, multi-step protocols involving clearing agents hindered clinical translation [68]. A transformative leap was achieved with the self-assembling and disassembling (SADA) platform. SADA-based BsAbs self-assemble in vivo into tetravalent structures, enabling capture of multiple radiolabeled hapten molecules (e.g., IMP288, DOTA). Crucially, they subsequently dissociate for rapid renal clearance, allowing efficient two-step PRIT without clearing agents [70]. Utilizing versatile haptens compatible with various diagnostic (PET/SPECT) and therapeutic radionuclides, this system has shown compelling efficacy targeting antigens like GD2, A33, and HER2 [71,72]. Preclinical studies demonstrated potent antitumor activity and a favorable safety profile for this approach. In GD2-expressing xenograft models, the agent achieved tumor-to-normal tissue uptake ratios exceeding 10, histological cure rates of over 50%, and a 68% extension in survival, all at a well-tolerated dose of 296 kBq per mouse. These findings underscore its tumor-targeting capability and therapeutic potential for solid tumors [73,74,75].

### 3.2. Bioorthogonal Click Chemistry

The inverse-electron demand Diels–Alder (IEDDA) reaction represents a powerful bioorthogonal chemistry platform for PRIT. In this approach, a tumor-targeting vector (e.g., an antibody) is functionalized with tetrazine (Tz). After its accumulation and clearance from circulation, a radionuclide conjugated to a trans-cyclooctene (TCO) ligand is administered. The rapid, specific, and irreversible “click” cycloaddition between Tz and TCO in vivo enables precise in situ assembly of the therapeutic radiopharmaceutical directly at the tumor site, achieving exceptionally high tumor-to-background ratios without exogenous clearing agents. Preclinical studies robustly validate this approach. Combining the IEDDA system with β-emitters (e.g., ^177^Lu) or α-emitters (e.g., ^225^Ac, ^212^Pb) yielded tumor-to-blood ratios exceeding 10:1 (tumor-to-blood ratios above 5 are typically viewed as favorable for imaging, whereas ratios exceeding 10 are generally preferred for therapeutic efficacy) in models of colorectal, pancreatic, and breast cancer models. These values indicate that most circulating radioactivity had been cleared before the imaging or therapy dose was given, which would reduce the risk of bone marrow toxicity [50,76,77]. Early-phase clinical trials (e.g., NCT04106492) have begun to establish preliminary safety and feasibility [78].

### 3.3. ESP-PNA System

The ESP-PNA pretargeting platform is designed to overcome two major limitations in solid tumor therapy: poor tissue penetration of large antibodies and dose-limiting renal/hepatic toxicity. This system leverages small (2–20 kDa), high-affinity ESPs such as Affibody molecules and designed ankyrin repeat proteins (DARPins) as primary targeting agents [79]. Their small size facilitates rapid tumor penetration and blood clearance. The ESP is conjugated to one PNA strand, while its complementary strand is linked to the radionuclide chelator. Subsequent injection of the radiolabeled counterpart leads to rapid and stable hybridization in vivo, concentrating the radiation dose precisely at the tumor [79].

This platform has demonstrated remarkable efficacy in preclinical models. In biodistribution studies, the percentage of injected dose per gram of tissue, abbreviated as %ID/g, is the standard measure of tumor accumulation. Values exceeding 10 %ID/g generally indicate strong target engagement, and those above 15 %ID/g are often considered sufficient for β-emitter-based therapy in murine models. In ovarian and breast cancer studies, Affibody-PNA pretargeting achieved tumor uptakes as high as 19 ± 2 %ID/g, while reducing blood and kidney activity by approximately 50-fold and 2-fold, respectively, compared to directly labeled probes [80]. Similarly, DARPin-PNA systems increased tumor uptake 8-fold while drastically reducing uptake in kidneys, liver, and spleen [66]. These results confirm the platform’s exceptional ability to enhance tumor-specific delivery while sparing critical organs. A significant ancillary advantage is its favorable translational profile: ESPs can be produced cost-effectively at high yields in bacterial systems, and PNAs are accessible via standard solid-phase peptide synthesis. This manufacturability, combined with compelling pharmacokinetic data, positions ESP-PNA hybrids as a promising and practical strategy for advancing targeted radionuclide therapy in solid tumors.

Despite the remarkable preclinical progress described above, pretargeted radioimmunotherapy has seen no regulatory approval after three decades of investigation, hindered by protocol complexity, dependence on clearing agents, non-specific organ retention, and immunogenicity [67,68]. Even the most technologically promising platform—bioorthogonal click chemistry—must address challenges including linker stability optimization (particularly preventing TCO isomerization to the less reactive cis-isomer), standardization of radionuclide dosing, and product heterogeneity from random conjugation. Progress requires not only continued technical refinement but also rigorous clinical trial design, robust manufacturing capacity, and realistic assessment of translational timelines.

## 4. Theranostics: Integrating Imaging and Therapy for Precision Medicine

**Figure 3:**
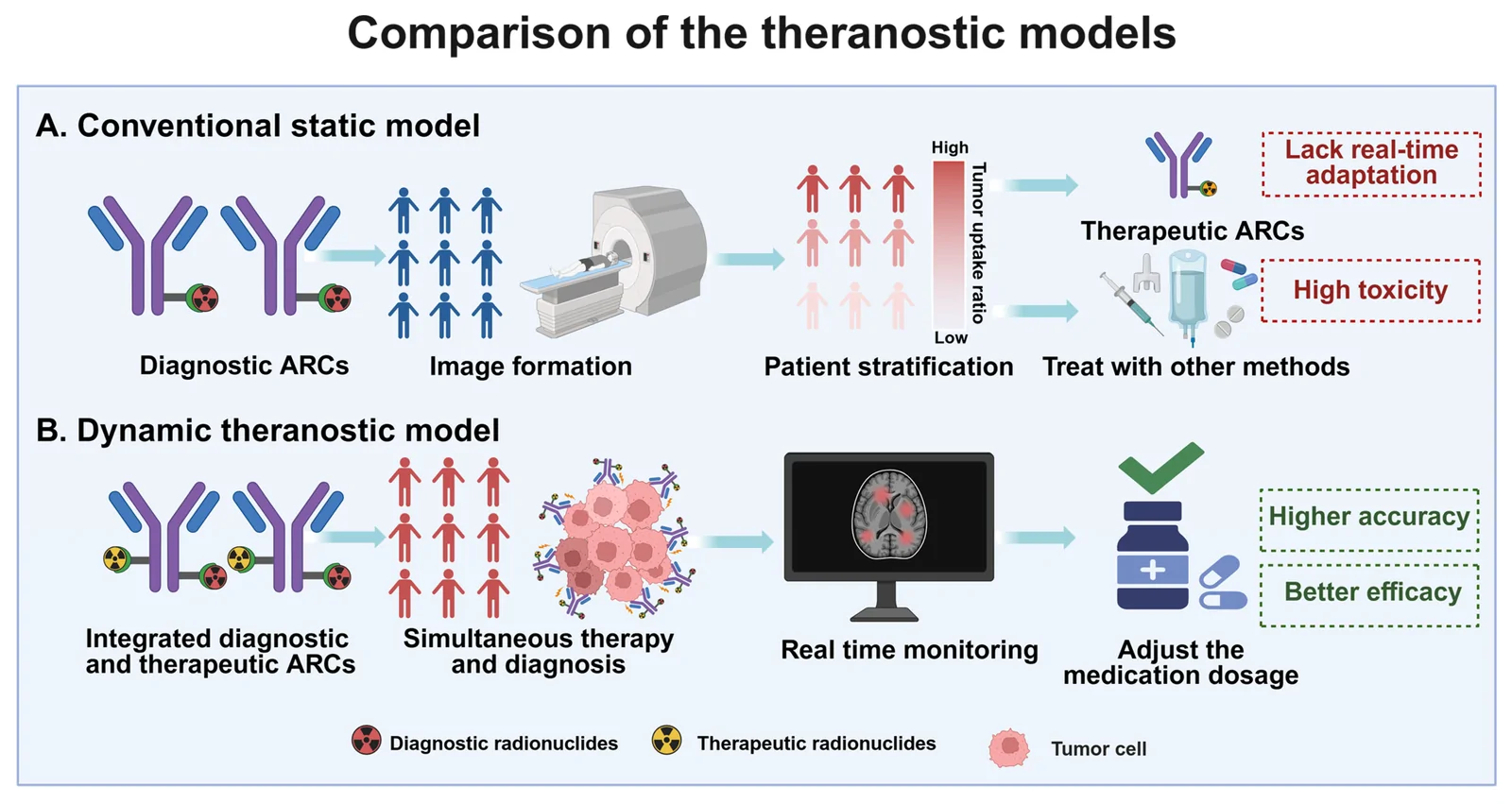
Comparison of conventional and integrated theranostic models for ARCs-based therapy. (**A**) Conventional static model: diagnosis and therapy are separate, sequential steps with a fixed treatment regimen. (**B**) Integrated dynamic model: diagnosis and therapy are linked in a closed-loop system, enabling real-time monitoring and adaptive dosimetry. Created in BioRender. mohan, Z. (2026) https://BioRender.com/214j1q5.

Overcoming the therapeutic bottlenecks of ARCs in solid tumors requires more than potent targeted killing; it demands a precision-guided strategy that integrates diagnosis with therapy. Theranostics, defined as the seamless integration of diagnostic imaging and targeted radiotherapy, addresses this need by employing diagnostic radionuclides for pre-therapeutic patient stratification, target quantification, and pharmacokinetic assessment, thereby optimizing the therapeutic window from the outset. Molecular imaging is indispensable in this paradigm for ensuring efficacy while managing toxicity. By utilizing matched radionuclide pairs directed against the same target, theranostics enables early tumor detection, precise biological characterization, and efficient eradication within a single framework, significantly enhancing the precision and controllability of solid tumor management (Figure 3). Unlike ADCs, which are restricted to therapeutic delivery, ARCs can be paired with diagnostic isotopes for theranostic applications, enabling patient selection and real-time dose monitoring within a unified platform. Critically, this theranostic workflow does not bypass patient selection; diagnostic imaging with a tracer dose is performed first to confirm target expression and favorable biodistribution, and only eligible patients proceed to therapeutic dosing. Mid-treatment monitoring then provides feedback for dose adaptation, completing the closed loop.

Matched radionuclide pairs form the basis of precision theranostics. An ideal pair requires similar pharmacokinetics between diagnostic and therapeutic nuclides to ensure that imaging accurately reflects therapeutic distribution. Recent progress in antibody-based systems has demonstrated promising results with multiple matched pairs. Among these, ^89^Zr-based pairs are the most widely applied. ^89^Zr (t_1_/_2_ = 78.4 h, β^+^) matches antibody pharmacokinetics and serves as the gold standard for immunoPET. The classic ^89^Zr/^90^Y combination pairs ^89^Zr for PET imaging with ^90^Y (t_1_/_2_ = 64.1 h, β^−^) for therapy; their closely matched half-lives and similar intracellular retention following internalization make them well-suited for antibody-based theranostics. Labadie et al. labeled an anti-GPC3 antibody with ^89^Zr and ^90^Y via DFO and DOTA chelators, respectively, enabling both PET imaging and radioimmunotherapy in hepatocellular carcinoma models [81]. The chemically distinct but more widely used ^89^Zr/^177^Lu pair combines ^89^Zr for imaging with ^177^Lu (t_1_/_2_ = 6.6 d, β^−^) for therapy. Despite their different coordination chemistries, their biodistribution aligns well in the context of slow antibody kinetics. This platform has been validated across multiple targets: ^89^Zr/^177^Lu-labeled anti-EphA2 antibody achieved high tumor uptake and significant efficacy in fibrosarcoma models [82], while ^89^Zr/^177^Lu-labeled amatuximab, an anti-mesothelin antibody, demonstrated synergistic immunoPET imaging and radioimmunotherapy in pancreatic cancer [83].

In addition, element-matched pairs offer superior pharmacokinetic concordance through identical coordination chemistry. The copper pair ^64^Cu/^67^Cu is a representative example. Using the sarcophagine chelator, researchers developed [^64^Cu]CuSar-trastuzumab for PET imaging and [^67^Cu]CuSar-trastuzumab for therapy. In HER2-positive tumor models, the ^64^Cu-labeled probe yielded high-quality PET images with prolonged tumor retention, while the ^67^Cu-labeled counterpart demonstrated potent therapeutic efficacy without observable radiotoxicity, offering a theranostic option for trastuzumab-resistant breast cancer [84]. Another element-matched pair, ^86^Y/^90^Y, combines ^86^Y (t_1_/_2_ = 14.7 h, β^+^) for PET imaging with ^90^Y (t_1_/_2_ = 64 h, β^−^) for therapy. Applied to ALT836, a monoclonal antibody targeting tissue factor, in pancreatic cancer models, ^86^Y-DTPA-ALT836 enabled PET imaging to guide subsequent ^90^Y-DTPA-ALT836 radiotherapy, which significantly inhibited tumor growth [85].

Emerging pairs continue to expand the theranostic frontier of ARCs. The ^133^La/^225^Ac pair combines ^133^La (t_1_/_2_ = 3.9 h, β^+^) for PET imaging with the alpha-emitter ^225^Ac (t_1_/_2_ = 9.9 d) for therapy. Using a macropa chelator for room-temperature labeling to avoid denaturation of heat-sensitive antibodies, this pair was applied to EGFR-targeted single-domain antibodies. PET imaging and biodistribution studies confirmed the in vivo concordance of this theranostic pair, providing a feasible pathway for alpha-based ARCs theranostics with temperature-sensitive antibody formats [86].

However, operationalizing this theranostic vision in routine clinical practice faces several practical bottlenecks that are often glossed over in proof-of-concept studies [87]. Standardization of site-specific conjugation represents a critical direction for clinical translation, as conventional random conjugation methods yield high product heterogeneity and poor batch-to-batch consistency [6]. Regarding nuclide pairing, theoretically ideal element-matched pairs (e.g., ^64^Cu/^67^Cu, ^152^Tb/^161^Tb) remain impractical for routine ARCs development in the near term due to extremely low production yields and prohibitive costs [6]. Furthermore, prospective validation of immunoPET-guided patient stratification in clinical trials is lacking, and a closed-loop decision framework integrating imaging, biomarkers, and therapy selection is urgently needed to advance ARCs theranostics from preclinical development to clinical practice.

## 5. Synergistic Combinations: ARCs and Immunotherapy to Remodel the Tumor Microenvironment

**Figure 4:**
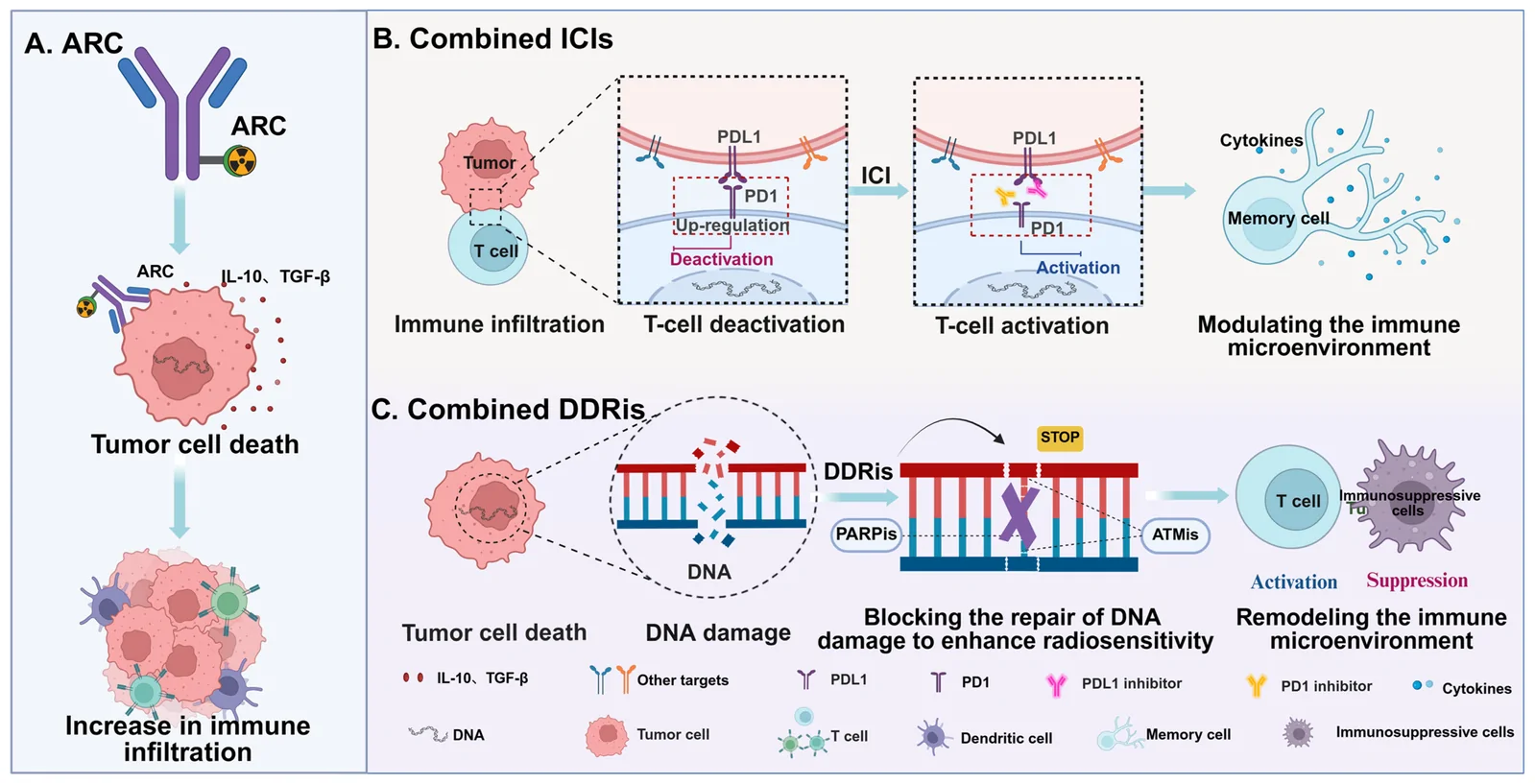
Mechanism of ARCs monotherapy and synergistic combined therapy. (**A**) Mechanism of ARCs in the treatment of cancer alone. (**B**) Combination with ICIs reactivates T-cell function and modulates the immune microenvironment. (**C**) Combination with DDRis blocks DNA repair pathways to enhance radiosensitivity and remodel the TME. Created in BioRender. mohan, Z. (2026) https://BioRender.com/kv0mukt.

The immunosuppressive TME of solid tumors, characterized by physical barriers and dysregulated cytokine networks, presents a formidable therapeutic challenge. Combining ARCs with immunotherapy represents a paradigm-shifting strategy to overcome this barrier. The combination of ARCs with immune checkpoint inhibitors (ICIs) holds potential to reverse immunosuppression, reactivate cytotoxic T cells, and foster long-term immune memory. Separately, co-administration with DDRis can induce radiosensitization and remodel the TME through selective blockade of DNA repair pathways. Together, these combinatorial approaches represent a promising translational direction for solid tumor treatment (Figure 4).

### 5.1. Combination with ICIs

The ARCs-ICIs combination is emerging as a cornerstone strategy to overcome resistance in solid tumors. This synergy operates through a cyclic mechanism: ARCs-mediated radiation induces immunogenic cell death (ICD), releasing tumor antigens and damage-associated molecular patterns that recruit and activate CD8^+^ T cells into the tumor bed. However, this activation often triggers compensatory upregulation of immune checkpoints (e.g., PD-1/PD-L1), leading to T-cell exhaustion [88]. Concurrently administered ICIs block these inhibitory signals, “releasing the brakes” on presensitized T cells. This “prime (ARCs) and release (ICIs)” cycle not only enhances immediate tumor killing but can also establish long-term immunological memory (Figure 4A) [89].

Robust preclinical evidence from multiple models supports this synergy. Zhao and colleagues employed ^177^Lu-labeled FAP-targeted LNC1004 with anti-PD-L1, achieving complete tumor eradication in MC38/NIH3T3-FAP models; rechallenged mice showed 100% tumor rejection. Mechanistically, single-cell RNA and T cell receptor (TCR) sequencing revealed reprogramming of the tumor immune microenvironment through enhanced CD8^+^ T cell activation, M1 macrophage infiltration, and TCR diversification [90]. In pancreatic cancer models, [^177^Lu]^177^Lu-LNC1004 combined with anti-PD-L1 produced complete regression within 40 days, while sequential ^225^Ac-LNC1004 followed by anti-PD-L1 cleared tumors in 6 of 9 mice over 90 days [91]. All regimens were well tolerated.

Beyond targeting tumor antigens, ARCs can directly eliminate immunosuppressive populations. Frank and colleagues combined ^225^Ac-anti-CCR8 with anti-CTLA-4 in colorectal cancer models. The combination yielded markedly stronger antitumor responses than either agent alone, driven by depletion of CCR8^+^ regulatory T cells and increased infiltration of CD8^+^ T cells, M1 macrophages, and NK cells. These findings confirm that ARCs-mediated targeting of tumor-infiltrating regulatory T cells (ti-Tregs) synergizes with checkpoint blockade through enhanced adaptive and innate immunity [92].

Clinically, ARCs-ICI combinations are advancing. ^177^Lu-girentuximab, targeting carbonic anhydrase IX expressed in over 90% of clear cell renal cell carcinomas, is under evaluation in the STARLITE 1 trial (NCT05663710) combined with nivolumab and cabozantinib in treatment-naïve patients. This strategy is hypothesized to activate cGAS-STING via DNA damage, thereby enhancing antitumor immunity and potentially improving response rates [93,94].

In prostate cancer, the PSMA-targeting antibody J591 has been evaluated in combination with pembrolizumab in a phase I/II trial (NCT04946370) for metastatic castration-resistant prostate cancer. A pooled analysis of 117 patients treated with ^225^Ac J591 demonstrated that baseline PSMA PET SUVmean (OR 1.13, *p* = 0.006) was significantly associated with PSA50 response, supporting the clinical potential of PSMA-targeted α-radioimmunotherapy combined with immune checkpoint blockade [95].

### 5.2. Combination with DDRis

Combining ARCs with DDRis offers a dual-pronged strategy: direct radiosensitization and immune modulation. Radiation from ARCs induces DNA damage, a lethal lesion that tumor cells counteract by activating DDR pathways. DDRis inhibit these repair mechanisms, enhancing ARCs’ cytotoxicity and overcoming radioresistance (Figure 4B).

#### 5.2.1. Poly(Adenosine Diphosphate-Ribose) Polymerase Inhibitors (PARPis)

PARPis, which block radiation-induced single-strand break repair, are the most clinically advanced DDRis for combination with ARCs. Mechanistically, this combination leverages synthetic lethality: PARPis impair DNA repair, while ARCs-delivered radiation creates additional lesions, together overwhelming tumor cell repair capacity. Dewulf and colleagues evaluated HER2-targeting single-domain antibodies labeled with ^131^I or ^225^Ac combined with olaparib in HER2-low xenograft models. Both radiolabeled constructs showed enhanced DNA damage and reduced cell viability with olaparib, and in vivo survival was significantly prolonged, confirming synergy [96]. In triple-negative breast cancer, anti-EGFR radioimmunotherapy combined with PARPis eradicated orthotopic tumors and metastases, an effect attributed to the elimination of cancer stem cells [97].

The clinical rationale for combining PARP inhibition with PSMA-targeted radionuclide therapy is increasingly recognized in mCRPC, where tumors with BRCA1/2 or ATM alterations are inherently sensitive to PARPis. A case report of a BRCA-mutant patient receiving ^177^Lu PSMA with short-course olaparib demonstrated a 90% PSA decline after a single cycle, far exceeding dosimetric predictions [98]. Independent phase III trials have established the survival benefit of olaparib (PROfound) and ^177^Lu-PSMA-617 (VISION) in molecularly selected mCRPC populations, providing a strong foundational rationale for their rational combination [99].

Collectively, these ARCs-based and broader radionuclide therapy findings establish a strong rationale for the clinical translation of PARPis combined with ARCs, with ongoing efforts now directed toward optimizing dosing schedules and identifying predictive biomarkers to guide patient selection.

#### 5.2.2. Ataxia Telangiectasia Mutated Inhibitors (ATMis)

ATMis, which inhibit the repair of the more lethal DNA double-strand breaks, represent a potentially more potent and immunogenic strategy than PARPis. Their mechanism is more directly aligned with the primary damage caused by radiation. Notably, ATMis possess unique immunomodulatory properties: they activate the cGAS-STING pathway, enhance antigen presentation on dendritic cells, increase CD8^+^ T-cell function, and reduce immunosuppressive cell populations [100,101]. In a prostate cancer model, ATMi combined with targeted radionuclide therapy demonstrated superior antitumor efficacy and higher complete response rates compared to PARPi combination, accompanied by enhanced TME remodeling through increased dendritic cell infiltration and activation. Unlike PARPis, which may upregulate immunosuppressive genes and promote T cell exhaustion, ATMis strongly activate STING signaling and suppress immunosuppressive pathways, thereby further potentiating antitumor immune responses [101]. In triple-negative breast cancer models, dual ENPP1/ATM inhibition heightened radiosensitivity, enhanced STING-TBK1 signaling, and induced robust innate and long-lasting adaptive antitumor immune memory, leading to significant tumor regression and abscopal effects [102].

Although clinically less mature than PARPis, ATMis such as AZD1390 are entering early-phase trials. AZD1390 is an orally bioavailable, highly selective ATM inhibitor optimized for blood–brain barrier penetration, making it particularly promising for combination with ARCs targeting brain tumors [103,104]. The combination of ARCs with ICIs or DDRis represents a multidimensional approach to overcome the immunosuppressive TME and radioresistance in solid tumors. While ARCs–ICI combinations are advancing clinically, ARCs-DDRis strategies offer a potent means of enhancing intrinsic radiosensitivity while concurrently modulating immunity [105]. Future directions include rationally designing combination sequences, identifying predictive biomarkers for patient stratification, and developing next-generation ARCs platforms optimized for synergy. These integrated strategies are poised to redefine the therapeutic landscape for advanced, refractory solid tumors.

Although the synergistic potential of ARCs with ICIs or DDRis is well-established in preclinical models, its clinical translation faces equally profound challenges. Clinical exploration of ARCs–immune checkpoint inhibitor combinations has revealed overlapping toxicities (myelosuppression, pneumonitis), unclear optimal sequencing, lack of predictive biomarkers, and limited translational value of murine models [88]. Moreover, the immunological effects of radiation are not uniformly activating; under certain dose rates and fractionation schedules, radiation may instead recruit myeloid-derived suppressor cells and regulatory T cells, thereby blunting the intended immune response.

## 6. Conclusions and Future Perspectives

ARCs have evolved from a simple concept of targeted irradiation into a multidimensional therapeutic platform that integrates precision delivery, real-time imaging, and synergistic immunomodulation. The preceding sections have detailed both the promises and inherent pitfalls of each technological pillar, from α-emitter physics and antibody engineering to pretargeting logistics and combination regimens. The path to widespread clinical adoption now demands not merely solving these isolated issues, but rethinking how they interconnect within a coherent translational framework. Looking forward, three overarching directions are poised to transform ARCs from a niche modality into a broadly applicable precision oncology tool.

First, the field is converging toward modular ARC platforms in which standardized components can be interchangeably assembled with diagnostic or therapeutic radionuclides. Site-specific conjugation, bioorthogonal click pairs, and engineered scaffolds have each demonstrated technical feasibility. The outstanding challenge lies in integrating these components into standardized, GMP-compliant platforms, particularly for multistep pretargeted regimens that require coordinated stability of click pairs (for example, tetrazine/TCO) and alignment with regulatory expectations. A practical priority is to establish industry-wide quality standards for these platforms and initiate early dialogue with regulatory agencies, thereby avoiding last-minute mismatches between product characteristics and approval requirements.

Second, the shift from empirical dosing to data-driven adaptive theranostics will be pivotal. Matched radionuclide pairs and multifunctional chelators have provided the necessary tools. The challenge now is embedding them into closed-loop workflows in which pretherapeutic imaging quantifies target occupancy and interpatient heterogeneity, while midtreatment dosimetric feedback dynamically guides dose adjustment or combination partner selection. A pragmatic entry point is to incorporate mid-treatment dosimetric feedback into existing basket trial designs, generating the evidence base for imaging-guided adaptive protocols in the near term.

Third, biomarker-guided precision synergy will be essential for optimizing combination strategies. Several biological rationales have been proposed to guide patient selection. For example, homologous recombination deficiency may identify patients likely to benefit from PARPi ARC combinations, while CD8^+^ T-cell infiltration or PD-L1 expression may predict response to ICI-ARC regimens. However, prospective validation of these biomarkers remains limited. A practical step forward would be to embed prespecified biomarker stratification into trial protocols, along with systematic collection of pre- and post-treatment tumor biopsies. This would establish the evidence base needed for biomarker-guided allocation and reduce the risk that subtype-specific strategies are discarded based on negative results from unselected populations. Finally, while scientific breakthroughs will continue to expand the ARCs arsenal, their real-world impact hinges on resolving ecosystem-level barriers: sustainable production and equitable distribution of scarce α-emitters, harmonization of dosimetric standards for regulatory approval, and long-term safety surveillance for delayed hematological and renal toxicities. These are not glamorous questions, but they are existential ones.

In summary, ARCs are no longer merely a radiotherapeutic add-on; they represent a convergent platform where molecular targeting, digital imaging, and immuno-oncology intersect. By embracing adaptive theranostics, modular engineering, and biomarker-driven combination design, the ARCs field is well-positioned to establish ARCs as a central pillar of future precision oncology, where real-time imaging informs each therapeutic decision and targeted irradiation becomes a customizable, adaptable modality rather than a fixed, one-time intervention.

## 7. Critical View

In our view, the next phase of ARC development for solid tumors will be defined less by incremental improvements to individual components, such as higher affinity antibodies, novel chelators, or more potent radionuclides, and more by the establishment of compatibility rules that govern how these components interact as an integrated system. The critical path forward is not simply to generate more optimized parts, but to understand the quantitative relationships that determine whether a given combination will succeed or fail.

The prevailing paradigm, component substitution followed by empirical in vivo testing, implicitly assumes that upgrading a single element translates into overall therapeutic gain. Yet system performance is ultimately dictated by its weakest link. A high-affinity antibody, for instance, can paradoxically impair deep tumor penetration through the binding site barrier effect. Pretargeting, despite its conceptual elegance, has not obtained regulatory approval after three decades, largely due to the procedural complexity of multi-step administration. These examples illustrate that the bottleneck is not the performance of any single component, but the absence of quantifiable rules for matching components to each other. The scarcity of published negative results further compounds this issue, leaving the field without systematic knowledge of which combinations are unlikely to succeed.

We argue that a more productive direction is to establish, prior to designing new candidates, foundational quantitative data linking antigen expression levels, antibody formats, and achievable tumor retention or absorbed dose. Such data are largely absent from the current literature. If this perspective encourages the field to reexamine its underlying assumptions, shifting from isolated optimization to compatibility-driven design, it will have served its purpose as a distinctive contribution to the field.

## Data Availability

No new data were created or analyzed in this study. Data sharing is not applicable to this article.

## Abbreviations

The following abbreviations are used in this manuscript:

ARCs	Antibody-radionuclide conjugates
ADCs	Antibody-drug conjugates
cORR	Confirmed objective response rate
Claudin 18.2	CLDN18.2
ATMis	Ataxia telangiectasia mutated inhibitors
BsAbs	Bispecific antibodies
DARPins	Designed ankyrin repeat proteins
ESPs	Engineered scaffold proteins
ICIs	Immune checkpoint inhibitors
IEDDA	Inverse-electron demand Diels–Alder
PNA	Peptide nucleic acid
SADA	Self-assembling and disassembling
scFv	Single-chain variable fragments
TCO	Trans-cyclooctene
Tz	Tetrazine
VHH	Variable domains of heavy-chain antibodies
PRIT	Pretargeted radioimmunotherapy
PARPis	Poly (adenosine diphosphate-ribose) polymerase inhibitors
ICD	Immunogenic cell death
DDRis	DNA damage response inhibitors
TCR	T cell receptor
Ti-Treg	Tumor-infiltrating regulatory T cells
TME	Tumor microenvironment
PSMA	Prostate-specific membrane antigen
